# Virus detection light diffraction fingerprints for biological applications

**DOI:** 10.1126/sciadv.adl3466

**Published:** 2024-03-13

**Authors:** Tongge Li, Ning Yang, Yi Xiao, Yan Liu, Xiaoqing Pan, Shihui Wang, Feiyang Jiang, Zhaoyuan Zhang, Xingcai Zhang

**Affiliations:** ^1^ School of Electrical and Information Engineering, Jiangsu University, Zhenjiang 212013, China.; ^2^ John A. Paulson School of Engineering and Applied Sciences, Harvard University, Cambridge, MA 02138, USA.; ^3^ Institute of Livestock Science, Jiangsu Academy of Agricultural Sciences, Nanjing 210014, China.

## Abstract

The transmission of viral diseases is highly unstable and highly contagious. As the carrier of virus transmission, cell is an important factor to explore the mechanism of virus transmission and disease. However, there is still a lack of effective means to continuously monitor the process of viral infection in cells, and there is no rapid, high-throughput method to assess the status of viral infection. On the basis of the virus light diffraction fingerprint of cells, we applied the gray co-occurrence matrix, set the two parameters effectively to distinguish the virus status and infection time of cells, and visualized the virus infection process of cells in high throughput. We provide an efficient and nondestructive testing method for the selection of excellent livestock and poultry breeds at the cellular level. Meanwhile, our work provides detection methods for the recessive transmission of human-to-human, animal-to-animal, and zoonotic diseases and to inhibit and block their further development.

## Teaser

An efficient optical diffraction system for high-throughput detection of virus infection at the cellular level is developed.

## INTRODUCTION

Given the zoonotic origin of the virus, detection of viral diseases is not limited to humans (*1*, *2*). It is also important to monitor animals such as livestock or common pets such as dogs and cats, as close contact between humans and animals can easily spread the virus. The large number of reported pet infections with severe acute respiratory syndrome coronavirus 2 (SARS-CoV-2) virus around the world is evidence of the fact that animals have some influence in carrying or transmitting infectious viruses (*3*, *4*). By revealing the growth state of cells related to virus invasion in descendants or animals, cross-species transmission or overflow of virus can be detected and blocked in time (*5*, *6*). However, the traditional in vitro detection of the degree of virus stress has a long cycle and has many kinds of reagents, and it is difficult to automate the process, which will cause irreversible damage to cells after detection. At present, the commonly used instrument detection method is limited by the defect of detection means, which cannot realize the information extraction of specific cell samples. Therefore, our field real-time method provides a facile research idea for controlling and preventing the spread of viral diseases. (*7*).

Because of technical obstacles, clinical detection of viral diseases requires a lot of time and complex experiments for analysis and testing, resulting in a small range of target viruses detected or a relatively low quality of binary information generated (*8*, *9*). Wang *et al.* (*10*) proposed to explore the severity of disease by viral load, and the results showed that although there was a certain correlation between the two, the accuracy of identifying viral infection among various infections was low. Chen *et al.* pointed out in their study on SARS-CoV-2 infection that there is a lack of effective clinical methods to capture the extensive heterogeneity of COVID-19 disease, and cell culture–based research is very necessary in such studies (*11*). Lee *et al.* (*12*) revealed a powerful interaction between viral diseases and individual cells through comprehensive analysis of plasma metabolites and protein levels and single-cell multi-omics analysis collected in clinical diagnosis. Fraser *et al.* (*13*) explored the pathogenesis of the virus, analyzed the protein structure with the help of host cells, and developed corresponding protease inhibitors, laying the foundation for the research and development of drugs that selectively inhibit the virus. Therefore, virology research based on the cellular level can not only optimize the detection process but also reveal the infection process in essence and explore the virus inhibition methods (*14*, *15*).

The traditional detection methods of virus stress cell status can be divided into labeled method and unlabeled method. The main labeling methods include Methyl thiazolyl tetrazolium (MTT), 2,3-Bis-(2-Methoxy-4-Nitro-5-Sulfophenyl)-2H-Tetrazolium-5-Carboxanilide (XTT), and Cell Counting Kit-8 (CCK-8) testings, the principle of which is to distinguish dead and alive cells and count the number of cells, on the one hand, and detect certain substances in cells that are closely related to the health status of cells, on the other hand (*16*, *17*). Because of the infringement of markers on cells, it is impossible to monitor cell status at multiple time nodes in a long cycle, and new cell activity detection methods are developed along the trend (*18*). Since the phase distribution of transmitted light is closely related to cell body morphology, Schmitz *et al.* (*19*) proposed to measure cell activity through microscopic imaging and RNA screening, and to extract relevant information of the measured object. However, the microscopic imaging technology has a small field of view, which is difficult to meet the high-throughput cell sample imaging and information extraction. Epsztein *et al.* (*20*) used CA1 pyramidal cell discharge to evaluate cell activity based on small changes in potential. Simons *et al.* (*21*) regulated the process of cell signal exchange through electrochemical signals and evaluated and manipulated the exchange activity. Electrical or electrochemical signals require special electrodes to establish a culture environment to adapt to cell growth, the research cost is high, and the electrode performance is required. At this time, the advantages of nondestructive testing technology based on spectroscopy were further highlighted (*22*).

The status of cytotoxicity can only be accurately assessed based on the statistical analysis of a large number of samples. Therefore, lensless imaging technology, which not only can image cells to a certain extent but also has the characteristics of high throughput and high automation, has high application value (*23*, *24*). On the basis of deep learning, Jo *et al.* (*25*) simultaneously imaged all aspects of a complete biological system on multiple space-time scales, and this model has wide applicability on different types of cells. Adams *et al.* (*26*) realized lensless imaging of tissues in vivo through an optical phase mask, which produced diffraction patterns with high-contrast profiles and accurately reconstructed low-contrast images of the detected samples. Schermelleh *et al.* (*27*) developed a diffraction imaging technique that bypassed the diffraction limit by super-resolution microscopy to visualize subcellular tissues and promote the study of cell activity and structure. Therefore, lensless holographic technology breaks the defects of traditional microscopy and provides a microscopic imaging technology with both high resolution and wide field of view, which can meet the requirements of high throughput for cell activity evaluation (*28*). Its imaging area can be expanded hundreds of times, providing greater flexibility to adapt to the needs of different samples and application scenarios.

Here, we are developing biological applications of continuous and high-throughput cell-based virus light diffraction fingerprints for virus detection, which can be applied for the detection of viral diseases in humans, pets, and livestock, and the selection and breeding of excellent livestock and poultry species at the cellular level. On the other hand, it can also detect the hidden spread of human-to-human, animal-to-animal, and zoonotic diseases in time and inhibit and block their further development. A case in point, taking the light diffraction spectrum of virus-stressed cells, the diffraction fingerprint of virus-stressed cells was obtained by using the lens-free large-field diffraction imaging platform, and the fingerprint information was quantified to screen the highly resistant cells. First, the influence on the optical path was explored according to the morphological changes of cells infected with virus. The diffraction fingerprints of cells with different morphologies were different to some extent. Second, we found a fingerprint characteristic that can effectively reflect the degree of virus stress on cells. By using gray-level co-occurrence matrix (GLCM), two parameters defined by GLCM, contrast (CON) and inverse differential moment (IDM), quantize fingerprint features. Finally, an online location detection system for virus-stressed cells based on lens-free diffraction was developed and the detection results were clustered. Swine testicle cell line (ST) and porcine pseudorabies virus (PRV) were used to carry out virus infection experiments on cells, which were compared with other diffraction fingerprint quantification methods, and the MTT method was used to verify the experimental results. Our method is a spectroscopic nondestructive testing as compared with many current virus detection methods, which require chemical reagents and are destructive. Our method realized localized detection that a certain cell can be located being infected with the virus, while many other cells are detected at the same time. The analytical parameters set in this paper are systematically studied for the first time, which can achieve better classification effect.

## RESULTS

### Construction of experimental platform

**Fig. 1.**
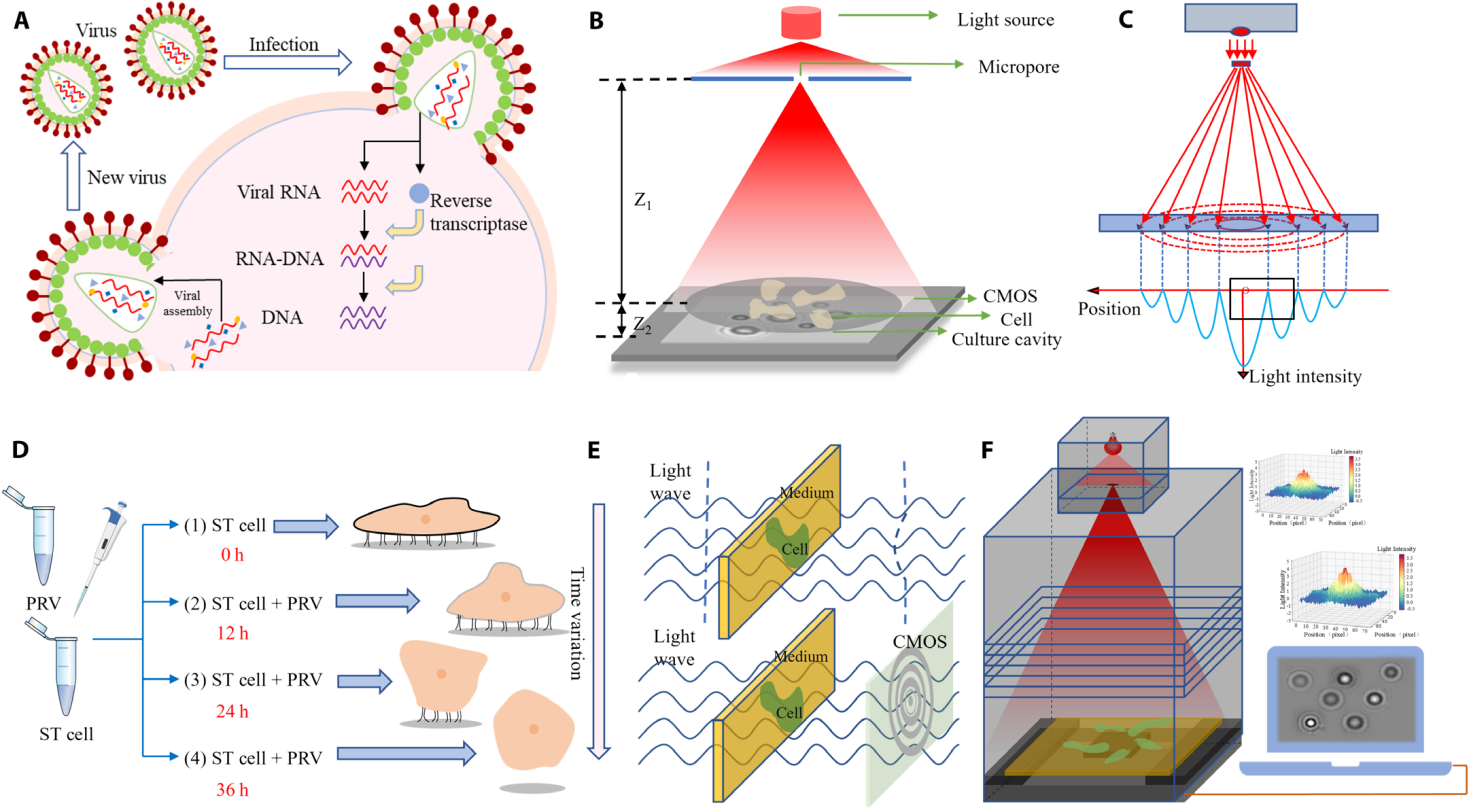
Principle and structure of the online location detection system for virus infection in cells based on lensless diffraction. (**A**) Change mechanism of cells after virus stress. (**B**) Schematic diagram of diffraction principles. (**C**) Principle of the diffraction fingerprint formed by the optical path in the diffraction process. (**D**) Schematic diagram of cell morphological changes at different times after virus stress. (**E**) Effect of different morphologies on the light path. (**F**) Schematic diagram of the device.

An online location detection system for virus-stressed cells based on lensless diffraction is designed, which consists of a large-field diffraction fingerprint acquisition platform and a computer. Its principle and structure are shown in Fig. 1. Figure 1A shows the change mechanism of cells under virus stress. Figure 1B is a schematic diagram of the diffraction principle in this paper. Figure 1C shows the principle of the diffraction fingerprint formed by the optical path in the diffraction process, that is, different positions show different light intensity; Fig. 1D shows the morphological changes of cells at different times after virus stress. Figure 1E shows that different morphologies of cells will affect the optical path and further generate different diffraction fingerprints; Fig. 1F is a schematic diagram of the device in this paper. The wide-field diffraction fingerprint acquisition platform is primarily composed of three components: a homemade diffraction box, a coherent light source, and an image sensor. A monochromatic light-emitting diode (LED) illumination lamp with a wavelength of 650 nm serves as the light source. Below the light source, a metal plate with microholes, measuring 300 μm in diameter, is centrally positioned. These microholes contribute to obtaining suitable light intensity and partially coherent light, thereby reducing interference. The light source is installed at the top of the diffraction box, and the height of the diffraction box can be manually adjusted to provide uniform illumination. This ensures real-time adjustment of the entire complementary metal-oxide semiconductor (CMOS) imaging area covered by the light. The light source is positioned approximately 6 cm above the sample plane. The culture dish is fixed on the sample platform above the CMOS to capture clear images within the imaging area. The distance between the bottom plane of the culture dish and the CMOS imaging plane is approximately 1 mm. We use a 5-megapixel CMOS image sensor with a pixel size of 2.2 μm, with an active imaging size of 5.70 mm × 4.28 mm, resulting in a large field of view coverage of up to 24.40 mm^2^. This field of view is 101.67 times larger than that of a conventional optical microscope at ×400 magnification. CMOS image sensors have a wider spectral response range and lower noise levels, providing sharper and more accurate imaging results. By expanding the sensitive area of the CMOS image sensor, the imaging field of view can be further increased. The resolution of the image capture lens is 1920 × 1080 dpi. The processor used is STM32H7, operating at a clock frequency of 480 MHz and a voltage of 3.3 to 5 V. It is connected to a computer via a full-speed USB (12 Mbs) interface to automatically record and capture diffraction images. The diffraction fingerprint’s texture features are extracted using the GLCM method, and the cell infection virus status is determined based on different features. It can be observed that the entire device is cost-effective and highly automated.

### Diffraction fingerprint analysis of cells with different morphologies

**Fig. 2.**
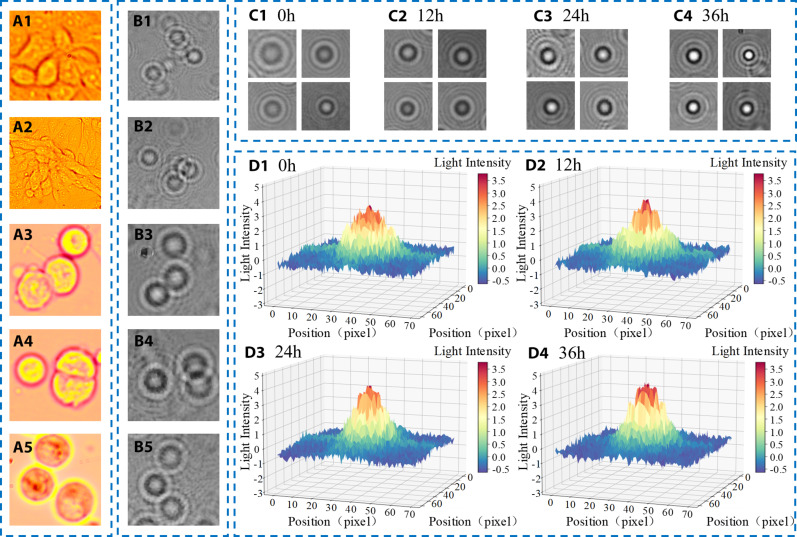
Diffraction fingerprint analysis of cells with different morphologies. (**A**) Changes in cell status after infection with the virus. (**B**) Diffraction fingerprints corresponding to different cell states. (**C**) Diffraction fingerprint morphology at different times of virus infection. (**D**) Relative three-position light intensity distribution corresponding to the diffraction fingerprint at different times.

In the virus-infected cell experiment, cell samples were treated with PRV and images were captured every 6 hours with a microscope, as shown in Fig. 2A. The results showed that cell morphology was closely related to the duration of virus infection, and cell morphology was closely related to the degree of virus infection. When there was no virus infection, the cells grew well; they were stretched and had a good flat morphology, attached to the bottom of the petri dish (see Fig. 2A1). Under the action of the virus, the cells are elongated, and the intercellular boundaries become clearer (see Fig. 2A2). Over time, cells become less active after being infected with the virus, and the cells contract, become round, and become less adherent, falling off the bottom of the petri dish and suspending in the medium (see Fig. 2A3 to A5). After that, diffraction fingerprints of cells in specific regions at different times were collected through the diffraction platform built in this paper, and Fig. 2B1 to B5 correspond to the diffraction fingerprints of Fig. 2A1 to A5, respectively.

It can be seen from the relationship between cell morphology and diffraction in the next section that cell morphology has a direct effect on the contrast of light and dark stripes of the diffraction fingerprint, and the difference of light intensity of light and dark stripes increases with the increase of cell thickness. After the cell was infected with virus, its shape gradually changed from flat to spherical, and the texture contrast and regularity of the diffraction fingerprint gradually became prominent. To confirm the feasibility of the theoretical analysis, the diffraction fingerprints of cells infected with virus at 0, 12, 24, and 48 hours were collected, and the grayscale processing was performed (see Fig. 2C1 to C4). Python software was used to calculate the relative light intensity of the diffraction fingerprints of cells with different coverage areas and shapes. By Franhofer’s diffraction formula:

$$E\left(t\right)=\frac{ke^{\mu z}}{z}2\pi a^{2}\left(\frac{z}{\mathrm{kaq}}\right)J_{1}\left(\frac{\mathrm{kaq}}{z}\right)$$

Therefore, the relative light intensity distribution of the diffraction fingerprint can be expressed as:

$$I=\frac{2k^{2}A^{2}}{z^{2}}{\left[\frac{J_{1}\left(\frac{\mathrm{kaq}}{z}\right)}{\frac{\mathrm{kaq}}{z}}\right]}^{2}$$

The results are shown in Fig. 2D, where Fig. 2D1 to D4 correspond to the relative light intensity of Fig. 2C1 to C4 diffraction fingerprints, respectively. To ensure the accuracy of the experimental results, the mean values of the four figures in Fig. 2C1 to C4 were calculated. The central relative light intensity of the diffraction fingerprints was the highest after the cells were completely dropped off, that is, 36 hours after infection with the virus. The results showed that with the increase of virus infection, the contrast of light and dark fringes was more prominent, the central brightness increased, and the central dark fringe was sharper, which was consistent with the theoretical analysis results. Therefore, it can be proved that the morphological changes of cells have a certain influence on the morphology of the diffraction fingerprint. Two distinguishing criteria of contrast and texture clarity are found through the analysis of the simulation results of relative light intensity, and then the contrast and contrast submatrix in the gray co-existence matrix are applied to further analyze the diffraction fingerprint.

### Diffraction fingerprint calculation and investigation

**Fig. 3.**
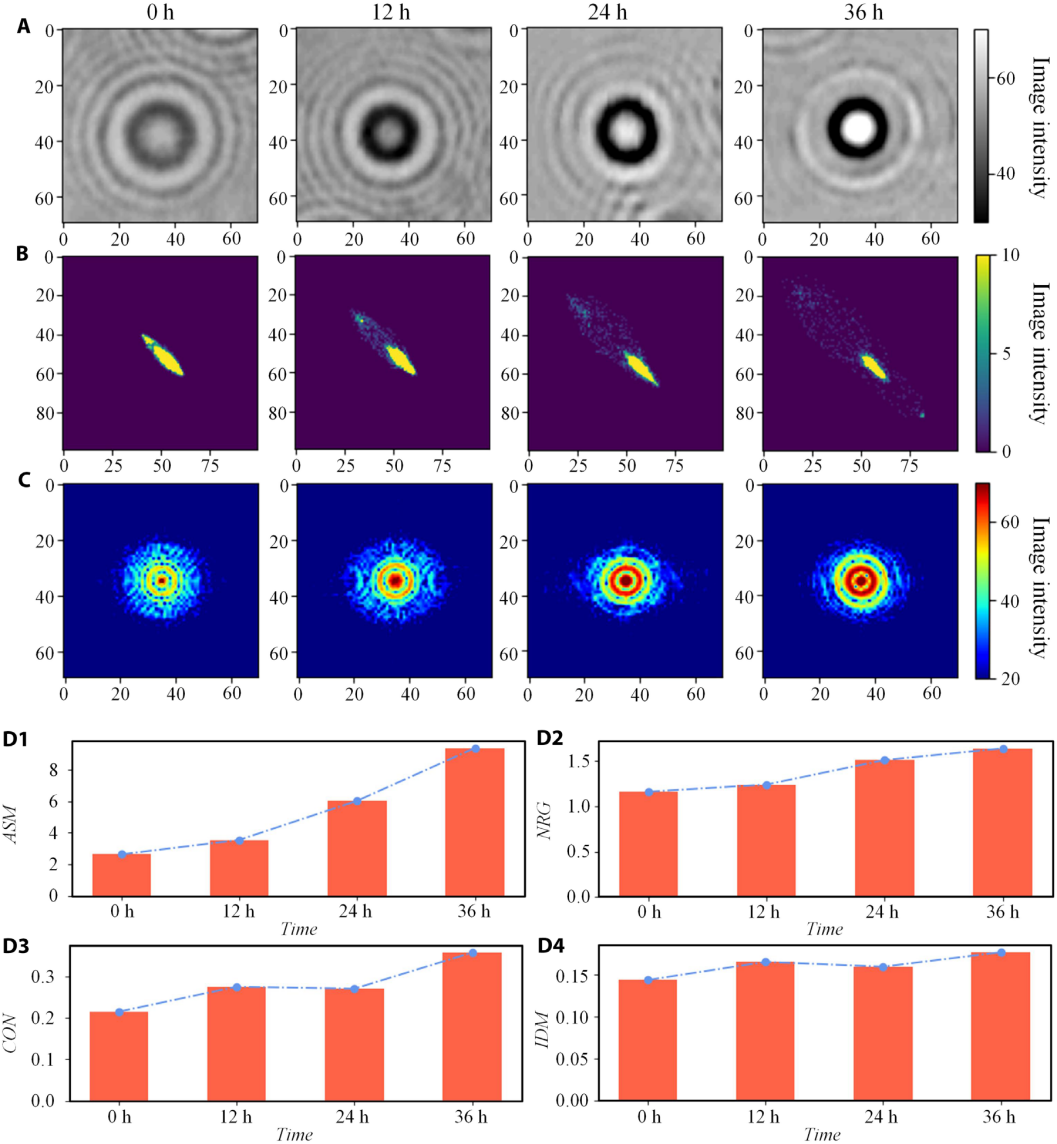
The gray co-occurrence matrix analyzes the diffraction fingerprints at different times of virus infection. (**A**) Diffraction fingerprint image after grayscale preprocessing and filtered by 3 × 3 mean filtering function. (The fingerprint image of 0 hours corresponds to the fingerprint in the lower left corner of Fig. 2C1. The fingerprint image of 12 hours corresponds to the fingerprint in the lower left corner of Fig. 2C2. The fingerprint image of 24 hours corresponds to the fingerprint in the lower right corner of Fig. 2C3. The fingerprint image of 36 hours corresponds to the fingerprint in the lower left corner of Fig. 2C4.) (**B**) Analytic diagram of gray co-occurrence matrix. (**C**) Two-dimensional light intensity map of diffraction fingerprint. (**D**) Quantified value of fingerprint characteristic [D1, angular second moment (ASM); D2, energy (NRG); D3, contrast (CON); D4, inverse differential moment (IDM)].

A fingerprint image was selected from the four cases in Fig. 2C to apply gray co-incidence matrix analysis, and the diffraction fingerprint was first transformed into gray image. Angles 0, 45, 90, and 135, pixel distance *d* = 1, and gray level 256/32 = 8 levels were used to create gray co-incidence matrix. Angular second moment (ASM), energy (NRG), CON, and IDM are selected to calculate the gray symbiosis matrix. Because different samples are taken manually, there are errors and noise under different observation conditions. Therefore, the gray fingerprint image in Fig. 2C is uniformly filtered by mean filter. We adopted 3 × 3 mean filtering, that is, the average addition of nine points, and the result after processing was shown in Fig. 3A. The final grayscale co-incidence matrix generated by the diffraction fingerprint in Fig. 3A is shown in Fig. 3B, and the fingerprint spectrum is shown in Fig. 3C. To observe the uniformity of gray distribution of diffraction fingerprint image and the depth of groove of texture, *ASM*, *NRG*, *CON*, and *IDM* of virus infection at 0, 12, 24, and 36 hours were shown, respectively. These four values used to quantify fingerprint characteristics and the calculation results are shown in Fig. 3D. The results show that the four features have obvious differences at different times.

The texture difference of light diffraction energy spectrum is our main research object. Of the four parameters, CON helps emphasize texture detail in an image and is widely used in texture analysis, object detection, and tasks that require distinguishing between different textures. To some extent, IDM has a certain effect on noise suppression. It considers the deficit outliers between pixel pairs, which helps to extract relatively smooth texture features in some noisy images. Therefore, in the subsequent classification process, CON and IDM parameters were selected as clustering indicators to comprehensively consider the details and smooth changes of textures in the fingerprint. On the one hand, it can improve adaptability and generalization; on the other hand, it can better deal with the influence of noise on texture features and get a better classification effect.

### Availability of diffraction parameters

**Fig. 4.**
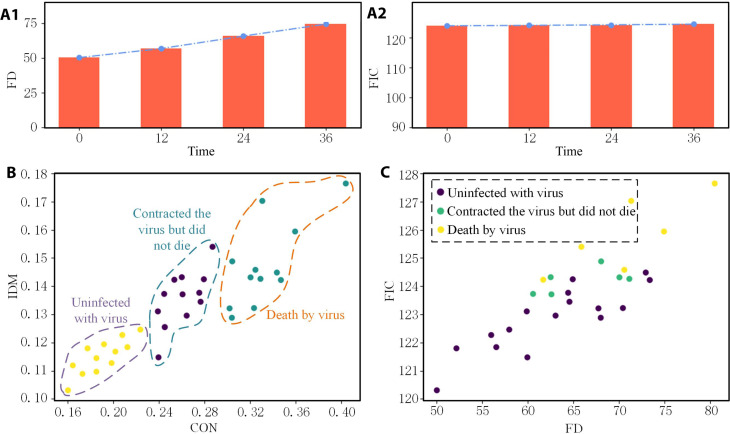
Clustering results and comparative analysis of diffraction fingerprints. (**A**) FD and FIC values of diffraction fingerprints [A1, fringe intensity contrast (FD); A2, fringe dispersion (FIC)]. (**B**) Sample partitioning results based on the proposed method. (**C**) Sample division results.

Two parameters, fringe intensity contrast (FIC) and fringe dispersion (FD), can be applied to evaluate cell activity.

$$\text{FIC}=\frac{\frac{1}{N}\sum_{O}{\left[I\left(x\right)-\bar{I}\right]}^{2}}{\sigma_{b}^{2}}$$

where *I*(*x*) is the pixel intensity of the *x*th pixel in the diffraction fingerprint of a single cell, $\bar{I}$ is the average pixel intensity of the diffraction fingerprint of a single cell, and σ_b_ is the SD of the background to eliminate the interference caused by background light noise, and the formula is as follows

$$\sigma_{b}=\sqrt{\frac{1}{M}\sum [{I_{b}\left(x\right)-\bar{I_{b}}]}^{2}}$$

where *I_b_*(*x*) and $\bar{I_{b}}$ are the *x*th pixel value and the average pixel value of the background image, respectively.

$$\text{FD}=\frac{\sum_{O}\left\{{\left[I_{n}(x)-I_{n}(O)\right]}^{2}∙d^{2}(x)\right\}}{\sum_{O}d^{2}(x)}$$

where *I_n_*(*O*) is the normalized pixel intensity of the centroid pixel, *d*^2^(*x*) is the distance between the *x*th pixel and the centroid pixel, and *I_n_*(*x*) is the normalized pixel intensity of the *x*th pixel in the diffraction fingerprint of a single cell, whose value is

$$I_{n}\left(x\right)=\bar{I}+\frac{I\left(x\right)-\bar{I}}{\text{max}\left(I\right)-\bar{I}}∙k$$

where *k* is a constant coefficient. To verify the accuracy of the proposed method, FD and FIC were calculated based on the 0-, 12-, 24-, and 36-hour diffraction fingerprints of virus infection collected in this paper, and the results were shown in Fig. 4A.

To use the collected fingerprint images for online evaluation of the duration of virus infection and cell status of the sample, 50 samples were collected for sample division and result evaluation using CON and IDM parameters based on the verification method in this paper, and FD and FIC parameters were used for sample division and result evaluation. Since the difference of CON and IDM parameters was small at 12 and 24 hours after infection with the virus, only 24 hours was taken as the representative time. Finally, the clustering results of the diffraction fingerprint samples were divided into three categories: not infected with the virus, infected with the virus but not dead, and dead after infection. Two samples were poorly differentiated, and the rest were successfully differentiated. The success rate of sample classification was 96%. The results are shown in Fig. 4, B and C, respectively. In Fig. 4B, different samples have clear boundaries, which can distinguish whether the samples are infected with the virus and determine the cell status. However, the method in Fig. 4C cannot achieve the classification effect.

On the basis of the analysis results, this method can easily distinguish infected and uninfected cells, effectively reveal the growth state of cells under the influence of viruses, and lay the foundation for the selection of excellent cells. Our method helps to discover the hidden transmission process of the virus between humans and animals, effectively inhibiting the spread of the virus. 

### Comparison and verification of results

**Fig. 5.**
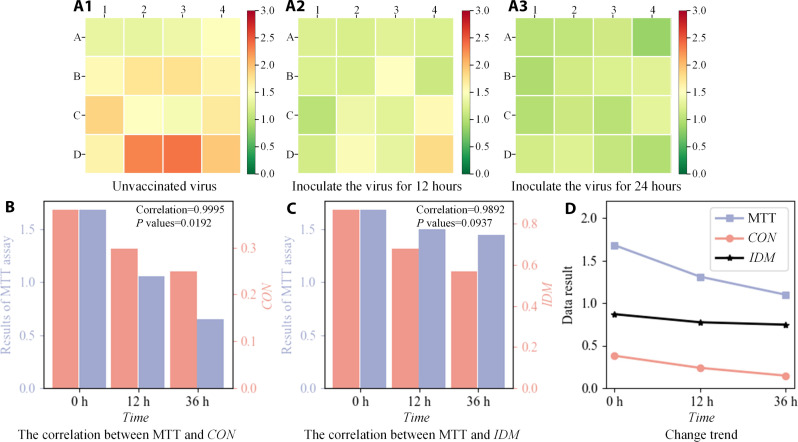
Comparison between the proposed method and MTT. (**A**) A1: MTT results without virus infection; A2: MTT results for 12 hours after inoculation with the virus; A3: MTT results for 36 hours after inoculation with the virus. (**B**) Correlation between CON value and MTT result. (**C**) Correlation between IDM values and MTT results. (**D**) Data change trend.

To evaluate the effectiveness of the proposed method, the same samples were compared by the MTT method, and ST cells were cultured in the medium for 24 hours until the cells were well attached. They were divided into three groups with 64 samples per group. Cells that are not infected with the virus are labeled group 1. Then, the cells were infected with 0.1% PRV and cultured for 24 and 36 hours, which were recorded as group 2 and group 3, respectively. The corresponding MTT results of groups 1, 2, and 3 are shown in Fig. 5A. To ensure the accuracy of the experimental results, each figure corresponds to 16 groups of data, and the average value of four culture cavities is taken for each group of data. With the increase of the virus infection time, the cell activity gradually decreases. To reflect the linear correlation between the detection method in this paper and the MTT method, the CON and IDM parameter values were compared with the MTT values, as shown in Fig. 5 (B and C). The correlation between our method and the cell viability measured by the MTT method was 98.9%. Figure 5D shows the linear graph of the three groups of data, and the linear correlation among the three groups of data can be intuitively seen. The correlation passed 99% significance test, and the *P* values of CON and IDM were 0.019 and 0.094, respectively. Therefore, the online virus infection detection method proposed in this paper has high accuracy, and the two parameters selected to quantify the diffraction fingerprint feature have high usability.

To detect the viral infection of the captured cell fingerprints online, the cells were cultured in 96-well plates for a specific time, the lensless diffraction information was extracted by a self-made device, and the diffraction fingerprint information was processed based on a customized evaluation index. The steps are as follows: (i) The 96-well plate is placed on the sample detection platform, and the culture plate is moved so that the samples at different infection times are successively located in the imaging area, that is, the sample area to be detected is in the same straight line with the light source, the microhole, and the CMOS. (ii) The diffraction fingerprint image of the sample is captured through the CMOS sensor and transmitted to the PC end. (iii) The diffraction fingerprint image is read, and the unique cell diffraction fingerprint corresponding to each cell from the entire diffraction image is extracted. (iv) The grayscale co-occurrence matrix is used to analyze the diffraction fingerprint, calculate the two parameters CON and IDM, apply the parameter values to the set clustering algorithm for classification, and determine the duration of virus infection of the cells according to the cell state.

This method is in good agreement with the evaluation results obtained by the MTT method used in related studies. In theory, the evaluation results of this method are better than the MTT method, because the MTT method cannot realize continuous detection of the same group of samples, which will lead to differences between samples. The method in this paper is continuous detection of the same group of samples, and its change trend can more accurately reflect the change of the state of the same group of samples with time after virus stress. The comparison results of the two methods are shown in Table 1. In the experimental process, compared with the MTT method, the reagents and consumables in this method are reduced, and no additional processing is required in the single detection process, which takes about 10 min to collect imaging and perform automatic analysis. However, the MTT method requires reagent treatment and chemical reaction lasts for 4 hours, and the advantages of this method will be more obvious when there are more types of doses to be detected. At the same time, the detection equipment of the MTT method is the enzyme label instrument, the general price is about $3000, and the diffraction imaging platform built in this paper is less than $300. This method has obvious advantages, and the detection results are in good agreement with the gold standard method, which can play an advantage in excellent cell breeding and blocking virus transmission.

**Table 1.** Comparison of virus detection at cell level by light diffraction fingerprinting with MTT, a gold standard assay for cell survival state.

Method	Equipment cost	Detection duration	Detection process
MTT	About $3000	About 40 hours	Reagent combination experiment
This paper	About $300	About 2 hours	Automate

## DISCUSSION

Virus is one of the important environmental factors that cause biological diseases. By revealing the growth state of cells related to virus invasion in organisms, cross-species transmission or overflow of virus can be detected and blocked in time. However, the traditional in vitro detection of virus infection has a long cycle and a variety of reagents, and the process is difficult to be automated, which will cause irreversible damage to cells after detection. At present, the commonly used instrument detection methods are limited by technology, require a lot of time and complex experiments for analytical testing, and cannot realize the information extraction of specific cell samples. Therefore, the new on-site real-time detection method is of great significance for controlling and preventing the spread of viral diseases.

We present a method for detecting viral infection based on light diffraction spectroscopy of virus-stressed cells. Here, we studied the effect of cell morphological changes on the optical model after virus stress and explored the relationship between cell state and light diffraction spectrum at different times of virus infection. The two parameters CON and IDM in the grayscale co-incidence matrix were selected to quantify the fingerprint characteristics, and the two parameters were verified to identify the virus-infected cells. ST cells were infected with PRV, the system was applied to obtain the diffraction fingerprint of the sample continuously, and the infection status and degree of the specified cells were judged by online algorithm. The reliability of this method in identifying virus infection was verified by comparing with existing methods of cell viability detection.

Our research provides an innovative viral detection method that eliminates the need for professionals in viral infectious disease prevention and cytovirology research and expensive complex specialized field detection equipment. The cost of the built platform is much lower than other platforms, no additional operation is required during the test process, and the sample is still operable after the test. Our continuous and high-throughput cell-based virus fingerprint research can be applied for the detection of viral diseases in humans, pets, and livestock, and the selection and breeding of excellent livestock and poultry species at the cellular level. On the other hand, it can also detect, inhibit, and block the further development of viral diseases in time. Our method is a spectroscopic nondestructive testing as compared with many current virus detection methods, which require chemical reagents and are destructive. Our method realized localized detection that a certain cell can be located being infected with the virus, while many other cells are detected at the same time. The analytical parameters set in this paper are systematically studied for the first time, which can achieve better classification effect.

Combining information from multiple fields, such as genomics, proteomics, and cytology, our method will allow more accurate models to be built (*29*–*33*). Further improvement in the accuracy and efficiency of virus disease detection will lay a solid foundation for the early screening of biological breeding. It can also prevent the spread of viral diseases in time and ensure the safety of human and animal life by itself or with other advanced technologies developed (*34*–*41*).

## MATERIALS AND METHODS

### Determination of wavelength and distance

The wavelength is a parameter of the diffraction transfer function, and the spectral characteristics are related to the coherence length, which reflects the temporal coherence of the light wave. Therefore, spectral characteristics are closely related to imaging. We analyze the influence of spectral distribution characteristics on imaging in terms of wavelength and coherence length. First, the imaging of cell samples under the same field of view under four different wavelength parameters is calculated by simulation. It is found that when the wavelength is longer, the diffraction fingerprint area is larger and the diffusion is stronger. But longer wavelengths accentuate overlap, while smaller wavelengths are not conducive to adequate interference. Second, the effect of coherence length in optical properties on imaging results is analyzed. The relationship between coherence length and spectral characteristics is $L_{c}=\lambda_{0}^{2}/\Delta \lambda$ , where *L_c_* is the coherence length, λ_0_ is the central wavelength, and Δλ is the maximum half-wave width. In theory, when the distance between two waves exceeds the coherence length, there is no stable interference relationship and no observable interference fringe can be formed. Therefore, this requires the coherence length of the light source to be greater than the fluctuation of the object to be measured. A red LED with a wavelength of 650 nm has a coherence length of 23.3 μm and a maximum half-wave width of 17.3 nm. We take the cell as the experimental object. Its own fluctuation is generally 5 to 30 μm. Through theoretical calculation and experimental investigation, the red LED light source with a wavelength of about 650 nm has better performance.

For the light source distance, when *Z*_1_ is small, the light intensity distribution on the recording surface is unbalanced, and when *Z*_1_ is large, the amplitude distribution is unbalanced. When two points on the surface of the sample meet *r*_1_ − *r*_2_ << *r*_1_, the amplitude at the two points is approximately equal. When any two points in a region of diameter *D* satisfy this condition, the incident light in the region can be approximated as a plane wave. According to the calculation, when *Z*_1_ > 57.7 mm, the amplitude is approximately evenly distributed in the area with a diameter of 1 mm on the recording surface, while the area covered by the adherents is generally several to tens of micrometers, and the area of the area far exceeds the area covered by a single cell. Therefore, for a microscopic cross section of a single cell, the incident light can be approximated as a plane wave when the light source is more than 6 cm away. However, when *Z*_1_ further increases, the intensity of the light field received per unit area in the sample region will decrease. The weak light field intensity is not conducive to obtaining higher quality imaging. For the image distance, *Z*_2_ is one of the parameters of the diffraction transfer function, which determines the propagation result of the diffractive light field. In the experiment, for a cell with a linearity of about 20 μm, the maximum linearity of the imaging region at the object image distance of 3 mm is about 100 μm. At this time, when the object image distance is doubled, the linearity of the imaging coverage area will directly be around doubled. On the basis of the above factors, the distance of light source is determined as *Z*_1_ = 7 cm, and the distance of object image is determined as *Z*_2_ = 3 mm.

### Cell culture and virus inoculation

By inoculating PRV into ST cells, recording the cell states at different time points, and collecting diffraction fingerprints, the performance of the proposed method was verified. Investigating the effects of viral infection on cells contributes to a better understanding of the changes in cell states after ST cell infection with PRV, allowing for the estimation of infection conditions and duration. The ST cells and PRV used in the study were obtained from the Jiangsu Academy of Agricultural Sciences in China. ST cells were cultured in Dulbecco’s modified Eagle’s medium (DMEM)/high-glucose medium (X-Y Biotechnology) supplemented with 10% fetal bovine serum (Capricorn) and 1% mixed solution of penicillin and streptomycin (X-Y Biotechnology). The cells were incubated in a water jacket CO_2_ incubator (Heal Force, HF160W) at 37°C in an atmosphere with 5% CO_2_ and 99% relative humidity. The ST cells were cultured for 48 hours to allow for adherence, and fresh culture medium was replaced every 24 hours. Cells were detached using 0.25% trypsin-EDTA and continued to be cultured in homemade culture dishes. The surrounding area of the culture dishes was three-dimensionally (3D) printed using transparent photosensitive resin material, and the bottom of the culture dishes was made of quartz (JGS1). The culture area was 25 cm^2^, and a total of 16 culture dishes were used in the experimental process, divided into four groups with four dishes each. An equal amount of ST cells was injected into the four groups of culture dishes. The first group of culture dishes was not inoculated with the virus and was labeled as the 0-hour virus inoculation. PRV was diluted 10^−3^ times the original concentration and then inoculated into the detached ST cells. The culture continued. In the second group, after 12 hours of virus inoculation, the four culture dishes were rinsed with phosphate-buffered saline (PBS), and the cell states and diffraction fingerprints of adherent cells were observed. At this time, the cell growth state was good, but elongation of cells and visible gaps between cells were observed. In the third group, after 24 hours of virus inoculation, the cell states and diffraction fingerprints were observed. At this time, some cells had partially detached but still exhibited adherent growth. In the fourth group, after 36 hours of virus inoculation, the cell states and diffraction fingerprints were observed. At this time, the cells were almost completely detached, indicating a significant cell death. The process of cell death after viral inoculation is accompanied by morphological changes. Therefore, exploring the relationship between cell morphology and diffraction can further analyze the corresponding viral infection conditions and duration indicated by diffraction fingerprints.

### The relationship between cell morphology and diffraction

After viral stimulation, adherent cells exhibit specific morphologies at different stages. With the progression of viral infection, the originally flattened and well-spread cell bodies undergo morphological changes. The cell edges gradually develop wrinkles and contract, while the central region becomes more elevated. As a result, the overall shape of the cells becomes more spherical, with a reduced coverage area. The cells gradually lose adhesion, shrink in size, and eventually detach completely from the substrate, floating in the culture medium. Its morphological changes are shown in Fig. 1D. The change of cell morphology will directly affect the absorption and scattering of light, and then affect the light path, and generate different diffraction fingerprint spectra.

The difference in phase change at various points of the medium, that is, the phase shift, depends on the difference in optical path Δ*OPL* when the light passes through different locations. The optical path here corresponds to the distance traveled by light in the medium and can directly reflect the phase change of the incident light wave after propagation in the medium. Because of the lack of cell walls in mammalian cells, their cytoplasm appears colorless and transparent. During the diffraction experiment, the amplitude modulation of the incident light by the cell body can be neglected, and the cell can be approximated as a purely phase object with complete transparency. Phase modulation is a modulation technique where the phase of a carrier signal deviates from its reference phase in proportion to the instantaneous value of the modulation signal. Its effect mainly depends on the refractive index distribution within the cell and the path traversed by the transmitted light in the cell. Here, the modulation of the incident light by the sample can be considered as pure phase modulation, generating a phase shift ∆φ(*x*, *y*) without amplitude attenuation *a*(*x*,*y*) = 0. Compared to the region of pure culture medium, the scattering points corresponding to the cell-covered region have different optical path lengths (*OPL*), resulting in different phase contributions to the formed wavefront. Therefore, the transmittance function of the entire sample region can be represented as

$$t(x,y)=\left\{\begin{array}{cc} \text{exp}(j0), & (x,y)\notin c\\ \text{exp}[j∆\varphi_{s}(x,y)], & (x,y)\in c \end{array}\right.$$

where *c* is the cell-covered region, that is, the wavefront in the cell-covered region has a certain distortion and deformation. Suppose that the cell thickness at a point within the cell coverage region *c* is *h_c_*(*x*,*y*), the cytoplasmic refractive index is *n_c_*(*x*,*y*), the overall thickness of the sample is *h*, and the refractive index of the culture medium is *n_m_*. Then, the optical path *OPL*(*x*,*y*) corresponding to the transmitted light passing through the cell-covered region point (*x*,*y*) is different from the optical path *OPL*_0_ corresponding to the point passing through the pure culture medium region, and the optical path difference is as follows

$$\begin{array}{c} ∆\text{OPL}=\text{OPL}\left(x,y\right)-{\text{OPL}}_{0}=n_{m}\left[h-h_{c}\left(x,y\right)\right]\\ +n_{c}\left(x,y\right)h_{c}\left(x,y\right)-n_{m}h \end{array}$$

Relative to the pure culture medium region, the phase shift at this point is

$${∆\phi }_{s}\left(x,y\right)=\frac{2\pi }{\lambda }\Delta \mathrm{OPL}=\frac{2\pi }{\lambda }\left[n_{c}\left(x,y\right)-n_{m}\right]h_{c}\left(x,y\right)$$

The optical model of unstained adherent cultured mammalian cells was constructed as follows

$$t(x,y)=\left\{\begin{array}{cc} \text{exp}(j0), & (x,y)\notin c\\ \text{exp}\left\{j,[,\frac{2\pi }{\lambda },[,n_{c},(,x,,,y,),-,n_{m},],h_{c},(,x,,,y,),]\right\}, & (x,y)\in c \end{array}\right.$$

To verify the accuracy of the above optical principles based on cell morphology, the diffraction fingerprints corresponding to different cell morphology were measured in Fig. 2, and the relative light intensity of the diffraction fingerprints was calculated.

### Analysis of diffraction fingerprints by GLCM

Through the analysis of the relationship between cell morphological changes and diffraction fingerprint, it can be seen that different cell morphology has a certain difference on the influence of light path, that is, each cell corresponds to a unique diffraction fingerprint. As a spatial gray level dependence matrix, GLCM is a method of texture feature extraction based on statistics. The diffraction fingerprint is a kind of image with clear texture. Here, the grayscale co-occurrence matrix was applied to analyze the diffraction fingerprint, and then the morphological changes of cells were detected to predict the status and duration of infection of cells with virus. The elements in the gray co-occurrence matrix represent the joint distribution of gray levels of two pixels with a certain spatial position relationship. At a given spatial distance *d* and direction θ, the gray level starts with *i* (row), and the probability of appearing gray level *j* (column) (normalized frequency, that is, divided by the sum of all frequencies) constitutes the element *P*(*i*, *j*∣*d*, θ) of the gray co-occurrence matrix. Any point in the image (*x*,*y*) and a point that deviates from it (*x + a*, *y + b*) form a pair of points. Suppose the gray value of the point pair is (*f*_1_, *f*_2_), and suppose the maximum gray level of the image is *L*, then the combination of *f*_1_ and *f*_2_ has L*L species. For the whole image, the number of occurrences of each (*f*_1_, *f*_2_) value is counted and then arranged into a square matrix. Then, the total number of occurrences of (*f*_1_, *f*_2_) is normalized to get the probability *P*(*f*_1_, *f*_2_), and the grayscale co-occurrence matrix is generated.

GLCM is defined by calculating the joint probability density of two-pixel gray levels. The mathematical expression is as follows

$$\begin{array}{c} P\left(i,j,d,\theta \right)=\left\{(x,y),(x+a,y+b)\in N\times N|f(x,y\right)\\ =i,f(x+a,y+b)=j\} \end{array}$$

Fourteen statistical features can be extracted from the gray co-incidence matrix, and there are four main features related to the texture information of the diffraction fingerprint image through analysis: angular second moment (*ASM*), energy (*NRG*), contrast (*CON*), and inverse differential moment (*IDM*). Through these four numerical quantization fingerprint features, the mathematical expressions are as follows

$$\text{ASM}=\sum_{i=0}^{L-1}\sum_{j=0}^{L-1}\left[P^{2}\left(i,j,d,\theta \right)\right]$$

*NRG* is the square root of *ASM*. The values of *ASM* and *NRG* are the sum of squares of the elements of gray co-occurrence matrix, reflecting the uniformity of gray distribution and texture fineness of the image, that is, the higher the value, the more uniform the texture change.

$$\text{CON}=\sum_{i=0}^{L-1}\sum_{j=0}^{L-1}\left[{\left(i-j\right)}^{2}P^{2}\left(i,j,d,\theta \right)\right]$$

*CON* measures the grayscale change between the reference pixel and its neighbors. The more intense the local gray change on the image, the deeper the texture groove, the clearer the visual effect, and the greater the contrast. Therefore, contrast indicates the sharpness of the image and the depth of the grain furrow.

$$\text{IDM}=\sum_{i}\sum_{j}\frac{P\left(i,j\right)}{1+{\left(i-j\right)}^{2}}$$

*IDM* reflects the clarity and regularity of the texture. When the texture is clear, regular, and easy to describe, the value is larger.
